# Supplementary material for: Sorting at embryonic boundaries requires high heterotypic interfacial tension
Source: Nat Commun. 2017 Jul 31;8:157. doi: 10.1038/s41467-017-00146-x (PMC5537356; doi:10.1038/s41467-017-00146-x)
Supplement: Supplementary file 2 — Supplementary Software 1 [file 41467_2017_146_MOESM2_ESM.zip › PottsModel/SrcPottsModel/doc/model/ProtrusiveEnergyFunction.html]

ProtrusiveEnergyFunction


---


|  |  |  |
| --- | --- | --- |
| **Overview** \| **Package** \| **Class** \| **Use** \| **Tree** \| **Deprecated** \| **Index** \| **Help** | |  |
| **PREV CLASS**   **NEXT CLASS** | **FRAMES**    **NO FRAMES**     **All Classes** |  |
| SUMMARY: NESTED \| FIELD \| CONSTR \| METHOD | DETAIL: FIELD \| CONSTR \| METHOD |  |


---


## model Class ProtrusiveEnergyFunction

```
java.lang.Object
  model.ProtrusiveEnergyFunction
```

**All Implemented Interfaces:**: EnergyFunction

---

``` public class ProtrusiveEnergyFunction extends java.lang.Object implements EnergyFunction ```

---

**Nested Class Summary**

**Nested classes/interfaces inherited from interface model.EnergyFunction**

`EnergyFunction.Energy`


| **Field Summary** | |
| --- | --- |
| `private static java.lang.String` | `name` |


**Constructor Summary**

`ProtrusiveEnergyFunction()`


| **Method Summary** | |
| --- | --- |
| `double` | `getDeltaEnergy(Pixel s, Pixel t)` |
| `EnergyFunction.Energy` | `getEnergyType()` |
| `private static double` | `getHeadProtrusiveEnergy(double protrusionElasticity, double angle)` |
| `java.lang.String` | `getName()` |

**Methods inherited from class java.lang.Object**

`clone, equals, finalize, getClass, hashCode, notify, notifyAll, toString, wait, wait, wait`

**Field Detail**

### name

```
private static java.lang.String name
```


**Constructor Detail**

### ProtrusiveEnergyFunction

```
public ProtrusiveEnergyFunction()
```


**Method Detail**

### getEnergyType

```
public EnergyFunction.Energy getEnergyType()
```

:   **Specified by:**: `getEnergyType` in interface `EnergyFunction`

---


### getName

```
public java.lang.String getName()
```

:   **Specified by:**: `getName` in interface `EnergyFunction`

---


### getHeadProtrusiveEnergy

```
private static double getHeadProtrusiveEnergy(double protrusionElasticity,
                                              double angle)
```

---


### getDeltaEnergy

```
public double getDeltaEnergy(Pixel s,
                             Pixel t)
```

:   **Specified by:**: `getDeltaEnergy` in interface `EnergyFunction`


---


|  |  |  |
| --- | --- | --- |
| **Overview** \| **Package** \| **Class** \| **Use** \| **Tree** \| **Deprecated** \| **Index** \| **Help** | |  |
| **PREV CLASS**   **NEXT CLASS** | **FRAMES**    **NO FRAMES**     **All Classes** |  |
| SUMMARY: NESTED \| FIELD \| CONSTR \| METHOD | DETAIL: FIELD \| CONSTR \| METHOD |  |


---
